# Marked INR Elevation Associated with Cefazolin: A Case Report

**DOI:** 10.1177/00185787251405375

**Published:** 2026-01-05

**Authors:** Bryce Beckstrom, Richard Wong, Tiffany LaDow

**Affiliations:** 1 The University of Texas at Austin, TX, USA; 2 Baylor Scott & White Medical Center, Temple, TX, USA

**Keywords:** adverse drug reactions, reporting/monitoring, anti-infectives, blood, infectious diseases, medication safety

## Abstract

Cefazolin is not typically considered to be an antibiotic that can increase the risk of bleeding. Yet, rare cases of cefazolin independently inducing a vitamin-K-responsive coagulopathy have been described. We report a patient with methicillin-susceptible *Staphylococcus aureus* (MSSA) endocarditis who developed an abrupt and significant international normalized ratio (INR) surge on cefazolin in the absence of exposure to vitamin K antagonists or azoles. Although the administration of a single dose of oral vitamin K rapidly corrected the INR elevation, the INR rebounded to supratherapeutic range later in the hospital course and ultimately required a daily low-dose vitamin K regimen to suppress the INR elevation. Upon readmission, the patient’s INR remained normal while on the combination of cefazolin and vitamin K supplementation. This case underscores that cefazolin can precipitate a clinically meaningful, vitamin-K-responsive INR elevation—especially in patients with renal dysfunction and low vitamin K reserve—and that proactive monitoring and supplementation may be warranted. Prior reports and mechanistic data are reviewed.

## Introduction

Cefazolin is a first-generation cephalosporin used widely for definitive treatment of MSSA infections and is particularly effective in treating bacteremia and endocarditis.^1^ INR-dependent coagulopathy with cefazolin is generally attributed to its potential interactions with warfarin. However, cases have been reported where cefazolin monotherapy, in the absence of warfarin, has induced hypoprothrombinemia with prolonged prothrombin time (PT), INR, and, in some cases, bleeding.^2-5^ These laboratory abnormalities often necessitate vitamin K administration and may compel discontinuation or substitution of cefazolin. Although the mechanism for cephalosporin-associated hypoprothrombinemia is classically linked to agents with an N-methyl-thiotetrazole (NMTT) side chain, cefazolin lacks NMTT. Alternatively, the mechanism for cefazolin-induced coagulopathy may be due to the inhibition of the vitamin K epoxide reductase pathway by cefazolin’s methyl-thiadiazole (MTD) side chain and suppression of intestinal vitamin K production.^6-10^ Evidence focused specifically on cefazolin is limited; nearly all cases are diagnosed by temporal association with cefazolin exposure, improvement after cessation and/or vitamin K, and exclusion of alternate causes such as hepatic dysfunction, disseminated intravascular coagulation, or anticoagulant use.^2-5^ We report the case of a marked INR elevation involving cefazolin in the absence of warfarin in a patient with MSSA endocarditis which was corrected rapidly with vitamin K replacement and subsequently maintained on low-dose oral vitamin K supplementation.

## Case Presentation

A 52-year-old female was admitted with fever, hyperglycemia, and suspected sepsis. Initial evaluation revealed leukocytosis and acute-on-chronic kidney disease. Blood cultures grew MSSA, and echocardiography demonstrated mitral valve endocarditis with perivalvular extension. The neurology team noted scattered small intracranial hemorrhages consistent with septic emboli. The cardiothoracic surgery team considered surgical intervention for source control of the vegetation, but ultimately this was deferred due to the patient’s unstable condition and the risk of exacerbating the intracranial hemorrhage with perioperative anticoagulation.

Empiric vancomycin and piperacillin-tazobactam were transitioned to cefazolin early during the first week for targeted MSSA therapy, dose-adjusted for renal function (scheduled 2 g IV every 12 hours). Heparin for venous thromboembolism prophylaxis was intermittently held; there was no exposure to warfarin, direct oral anticoagulants (DOACs), azole antifungals, or amiodarone. Albumin levels during the admission were low (1.8-2.1 g/dL). There was no clinical or laboratory documentation of intrinsic hepatic disease during either admission (no evidence of chronic liver disease, and routine liver chemistries were not reported as abnormal).

On hospital Day 10 (Day 7 of cefazolin therapy), two INRs drawn hours apart were 8.6 and 8.7. Oral vitamin K (phytonadione) was ordered at 5 mg PO once and, by the following days, the INR fell to 1.3 to 1.5. Immediately following the INR measurements on hospital Day 10, a plasma vitamin K level was obtained at 0.17 nmol/L. A pre-albumin (transthyretin) level drawn the same day measured 11.6 mg/dL. Together, these results are consistent with reduced vitamin K reserves and impaired nutritional status at the time of INR elevation; they support the decision to administer vitamin K and subsequently initiate low-dose maintenance supplementation. The patient’s INR remained <2 for several days but climbed to 3.3 on Day 19; daily low-dose vitamin K1 (100 mcg PO) was started with stabilization, and the patient was discharged the next day on cefazolin with outpatient follow-up. Other coagulation parameters did not indicate an alternative coagulopathy (eg, disseminated intravascular coagulation) to account for the isolated INR rise; therefore, detailed values (PT/aPTT/fibrinogen/hemoglobin) are not presented.

**Figure 1.**
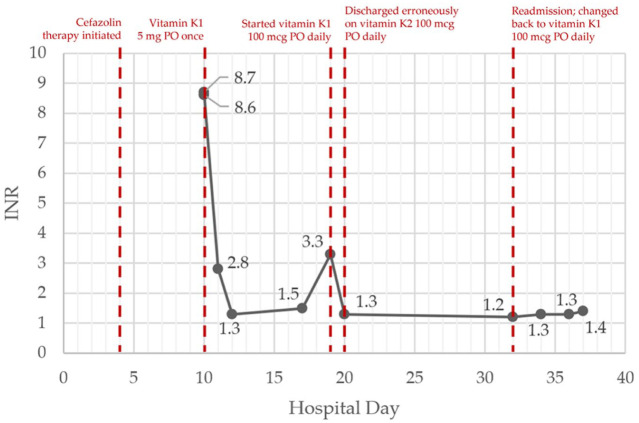
INR trend by hospital day and key interventions. This summarizes the INR trajectory by hospital day (days since first admission) across both admissions and highlights the timing of vitamin K interventions.

The patient returned for a second admission 12 days following her discharge. At intake it was recognized that the discharge prescription from the previous admission had been for vitamin K2 rather than vitamin K1. Despite that discrepancy, the admission INR was normal and remained between 1.2 and 1.4 on repeated testing while cefazolin was continued and vitamin K1 100 mcg PO daily was used instead of K2. Renal function remained reduced but stable; albumin on this second admission was 2.6 to 2.7 g/dL. The patient had no bleeding attributable to cefazolin. The INR trend by hospital day and key interventions are shown on Figure 1.

## Discussion

This case documents a reproducible, vitamin-K-responsive coagulopathy temporally associated with cefazolin in a patient with MSSA endocarditis, chronic kidney disease, hypoalbuminemia, and poor nutritional reserve—features that converge with risk factors emphasized in prior reports. Although cefazolin lacks the classical NMTT side chain, mechanistic and clinical data suggest its MTD (2-methyl-1,3,4-thiadiazole-5-thiol) leaving group can also perturb vitamin-K-dependent γ-carboxylation. In human and animal work, cefazolin and the liberated MTD moiety have been shown to inhibit the vitamin K cycle; the appearance of vitamin K epoxide after cefazolin exposure supports vitamin K epoxide reductase complex subunit 1 (VKORC1) pathway interference.^6,7^ Impaired dietary and microbiota-derived vitamin K2 supply may compound this, especially in malnutrition or with broad-spectrum antibiotics.^8-10^ In this patient, the low vitamin K concentration (0.17 nmol/L) and reduced pre-albumin on hospital Day 10 provide objective contemporaneous evidence of limited vitamin K reserve and poor protein-calorie status at the time of INR elevation, which plausibly increased susceptibility to cefazolin-associated coagulopathy. Additionally, the absence of documented intrinsic hepatic disease reduces the likelihood that liver dysfunction contributed materially to coagulopathy in this case. A causality assessment was performed using the Naranjo Adverse Drug Reaction Probability Scale, a standardized questionnaire that assigns a numerical score based on factors such as temporal relationship, rechallenge, alternative causes, and objective evidence. In this case, the total Naranjo score for the association between cefazolin and the marked INR elevation was 7, which falls within the range of 5 to 8 and therefore classifies the event as a “probable” adverse drug reaction attributable to cefazolin.

Our patient’s coagulation profile evolved in two phases: (i) a sudden INR surge on hospital Day 10 while receiving cefazolin, with prompt reversal after oral vitamin K; and (ii) a later rebound into supratherapeutic range, again vitamin-K-responsive (controlled with daily 100 mcg). The pattern is concordant with published cases in which INRs climbed during cefazolin therapy, improved rapidly with vitamin K, and stabilized after cefazolin cessation or supplementation. For example, Ngiam et al^2^ (MSSA endocarditis) reported INR elevation during cefazolin therapy in an older adult with malnutrition and kidney injury; INR normalized within ~48 hours after stopping cefazolin and giving oral vitamin K. Chung and Watson described hypoprothrombinemia after 7 days of cefazolin in postoperative renal failure; correction followed vitamin K and drug withdrawal.^3^ M. Smith et al^4^ presented a spontaneous retroperitoneal hemorrhage with markedly elevated INR on cefazolin—even in normal renal function—again resolving with vitamin K and discontinuation. More recently, R. Smith et al^5^ highlighted severe cefazolin-associated coagulopathy (hematochezia, prolonged PT/INR) that corrected after vitamin K and stopping cefazolin; noteworthy, the patient had confounding rivaroxaban exposure, but the coagulopathy persisted despite holding the DOAC until vitamin K and cefazolin cessation.^5^ Together, these cases emphasize that (a) cefazolin can raise INR even without warfarin, (b) malnutrition and renal dysfunction amplify risk, and (c) vitamin K (and often cefazolin cessation) rapidly reverses the defect.

In our case, cefazolin was continued because its potent bactericidal kill-curve and lower sodium content made it the optimal agent for MSSA endocarditis in a patient with heart failure, and daily vitamin K supplementation was chosen to blunt recurrent INR elevation. This approach aligns with older cephalosporin literature where prophylactic or reactive vitamin K was used in high-risk settings to prevent hypoprothrombinemia.^6,11^ The transient use of vitamin K2 (menaquinone) after discharge likely did not contribute to INR control; most clinical experience for reversal and prevention of cephalosporin-related coagulopathy involves vitamin K1 (phytonadione), the preferred substrate for hepatic γ-carboxylation.^9^ Low albumin and reduced renal clearance in our patient plausibly increased free cefazolin and extended exposure to its MTD metabolite, accentuating the effect. The consistent normalization of INR on the patient’s second admission while on cefazolin plus daily vitamin K supports a supplementation strategy when cefazolin is indispensable. Clinically, we recommend a baseline INR to be obtained in high-risk patients (on warfarin, renal impairment, malnutrition, poor intake/antibiotic-altered microbiota) prior to starting cefazolin. Serial INR monitoring is not routinely recommended, even in high-risk patients, due to the rarity of cefazolin-induced hypoprothrombinemia. If the patient develops signs or symptoms of an elevated INR (eg, bleeding), then serial INR monitoring should begin coupled with low-dose vitamin K supplementation and a clinical decision on the necessity of continuing cefazolin. If cefazolin is deemed necessary, then a daily low-dose vitamin K supplementation is recommended to prevent coagulopathy with individualized dosing based on response.

## Conclusions

Our patient adds another case where cefazolin monotherapy caused an abrupt INR elevation in the absence of warfarin. In literature, patients with renal dysfunction, low albumin/poor intake, or prolonged broad-spectrum antibiotic exposure appear at greater risk. In such patients, establishing a baseline INR prior to starting cefazolin is prudent. When INR rises, vitamin K effectively reverses coagulopathy. Importantly, while a single dose of vitamin K can rapidly correct the INR, a low-dose maintenance regimen (eg, daily supplementation) is needed if cefazolin is continued. If INR remains unstable or bleeding develops despite supplementation, alternative anti-staphylococcal therapy should be considered as the discontinuation of cefazolin should eventually normalize the INR. Further study is needed to define the incidence, refine risk stratification, and clarify the role of prophylactic vitamin K during prolonged cefazolin courses. This case report was prepared using the CARE reporting guidelines.^12^
